# Mechanism of bonding, surface property, electrical behaviour, and environmental friendliness of carbon/ceramic composites produced via the pyrolysis of coal waste with polysiloxane polymer

**DOI:** 10.1007/s11356-023-28661-z

**Published:** 2023-07-29

**Authors:** Orevaoghene Eterigho-Ikelegbe, Ryan Trammell, Emmanuel Ricohermoso, Samson Bada

**Affiliations:** 1 DSI-NRF SARChI Clean Coal Technology Research Group, School of Chemical and Metallurgical Engineering, Faculty of Engineering and the Built Environment, University of the Witwatersrand, Private Bag X3, Wits 2050, Johannesburg, South Africa; 2 Fachbereich Material-Und Geowissenschaften, Technische Universität Darmstadt, Otto-Berndt- Straße 3, 64287 Darmstadt, Germany; 3 Semplastics, 269 Aulin Avenue, Suite 1003, Oviedo, FL 32765 USA

**Keywords:** Circularity, Coal waste, Contact angle, Leaching, Recycling, Waste utilisation

## Abstract

A simple mixing-pressing followed by thermal curing and pyrolysis process was used to upcycle coal waste into high-value composites. Three coal wastes of different physicochemical properties were investigated. The hypothetical mechanisms of bonding between the coal particles and the preceramic polymer are presented. The textural properties of the coals indicated that the lowest volatile coal waste (PCD) had a dense structure. This limited the diffusion and reaction of the preceramic polymer with the coal waste during pyrolysis, thereby leading to low-quality composites. The water contact angles of the composites up to 104° imply hydrophobic surfaces, hence, no external coating might be required. Analysis of the carbon phase confirmed that the amorphous carbon structure is prevalent in the composites compared to the coal wastes. The dc volume resistivity of the composites in the range of 22 to 82 Ω-cm infers that the composites are unlikely to suffer electrostatic discharge, which makes them useful in creating self-heating building parts. The leached concentrations of heavy metal elements from the composites based on the end-of-life scenario were below the Toxicity Characteristic Leaching Procedure regulatory limits. Additionally, the release potential or mobility of the metals from the composites was not influenced by the pH of the eluants used. On the basis of the reported results, these carbon/ceramic composites show tremendous prospects as building materials due to these properties.

**Graphical Abstract:**

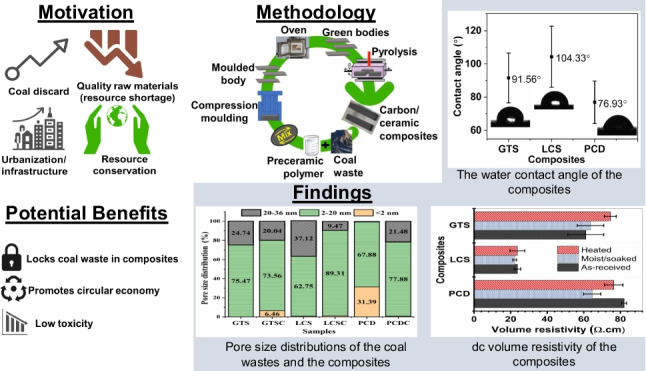

## Introduction

The beneficiation of run-of-mine coal to produce clean coal for power and metallurgical applications and mechanised mining generate coal waste. According to the National Development and Reform Commission of China, the country has already accumulated over 4.5 Gt of coal gangue, which is growing annually with an increase of 370 to 550 Mt (Yang et al. 2018). In South Africa, approximately 60 Mt of coal waste is produced annually with an accumulation already over 1 Gt (Belaid et al. 2013; Isaac and Bada 2020). Therefore, it can be anticipated that if the global coal demand flattens to around 7.4 Gt by 2025 as predicted by the IEA (IEA 2020), the amount of coal waste is likely to increase. Unfortunately, only a small proportion of this coal waste is processed into high-value, high-performance products. The remainder is traditionally disposed of in landfills or piled up in arable land, resulting in unintended health, safety, and environmental impacts. While interventions like remediation and reclamation solutions such as mine backfill have evolved significantly, these solutions leave behind risks and liabilities. In addition, their long-term effect is not guaranteed (Lottermoser 2011; Broadhurst et al. 2019; Zhang and Ling 2020). Therefore, given the enormous volume of coal waste, immense research attention is devoted to valorisation in line with the objectives of the circular economy model and sustainable development.

Significant research exists in the field of valorisation of coal waste, which includes the synthesis of porous materials for natural gas storage and energy storage, coal briquettes and pellets production, carbon fibre, rare earth extraction, etc. (Abdulsalam et al. 2019, 2020; Eterigho-Ikelegbe et al. 2021a, b; Harrar et al. 2022). Of these research fields, recycling coal waste into composites stands out because it is possible to consume huge quantities of coal waste to produce high-volume composites, preventing their accumulation. Furthermore, composite technologies have been at the forefront of developing advanced materials in recent years, resulting in considerable research attention. This is because composite technology allows the combination of different materials to form products of superior properties compared to products of unit material. Hence, most electronic components, megastructures, sports and packaging equipment, automotive parts, biomedical facilities, construction, etc., are composites (Mohanty et al. 2018; Rajak et al. 2019).

On the other hand, preceramic polymers (PCPs), otherwise known as silicon-based polymers, have been successfully explored to fabricate polymer-derived ceramic (PDC) parts and ceramic matrix composites for more than four decades (Colombo et al. 2010; Ionescu et al. 2010). The PCP route is preferably pursued to realise complex-shaped ceramic bodies and metastable inorganic network structures at relatively low processing temperatures (Colombo et al. 2010; Ding et al. 2012; Ricohermoso et al. 2022). The molecular structure, chemistry and heat treatment conditions of these polymers can be modified to produce custom-made amorphous or nanocrystalline microstructure PDC of interesting features (Bhandavat et al. 2012; Fonblanc et al. 2018; Shen et al. 2018). Ceramic parts produced via the PCP route include but are not limited to fibres, coatings, energy storage materials, films, monoliths, catalyst support, microelectromechanical systems, and so more (Mutin and Boury 2003; Colombo et al. 2010; Shen et al. 2018; Wen et al. 2020; Ricohermoso et al. 2021). The PCPs, polysiloxanes, are generally stable in air and humid atmosphere, making them easy to manipulate. These polymers are thermally stable, produce no toxic decomposition products, industrially available, and inexpensive to produce (Walter et al. 1996; Cordelair and Greil 2000). Shen et al. (2018) reported that the structure of the polysiloxane-derived ceramic contains nanodomains of silicon dioxide surrounded by carbon nanodomains. This attribute imparts the ceramic with unprecedented chemical and thermal stability, low density, and good mechanical properties.

The world’s population is expected to quadruple to more than 9.7 billion by 2050 (OECD 2015). As a result, there is an imminent depletion of building raw materials that are likely to become expensive due to high demand and high energy prices. The material-intensive building industry must, therefore, consider technological waste and composites as an effective strategy to address these challenges. Recently, an innovative approach for producing structural carbon/ceramic composites from two different US bituminous coal using the polysiloxane PCP as a binder for the coal particles was documented (Hill and Easter 2021; Sherwood et al. 2021). The investigators reported that the lower-grade coal yielded composites of higher linear shrinkage (19.4%) and lower char yield (68.3%). In contrast, the higher-grade coal yielded superior composites of linear shrinkage and char yield of 18% and 80%, respectively. The higher-grade coal contained low volatiles and was less absorbent to the PCP resin than the lower-grade coal. In terms of weight and strength, the higher-grade coal resulted in a lighter and higher-strength composite than the lower-grade coal. Furthermore, a fire test conducted using a propane torch could not cause significant damage to the composites, reflecting their excellent fire resistance. The positive inherent properties of the composites prompt their suitability as a prime alternative to building materials, such as conventional bricks, blocks, panels, roofing sheets, and even wood polymer composites.

But variations in the origin, vegetation, and climatic conditions at the time of coal formation, together with subsequent events in the geological history of coal coalification, give rise to coal with different compositions (Falcon 1986). Interestingly, most South African coals are bituminous rank (medium rank C) and are of low-sulphur content, but higher in ash and inert organic matter content (inertinite-rich) compared to the northern hemisphere coals (Falcon 1986; Hancox and Götz 2014). It is therefore important to match coal or coal waste for specific fields of utilisation because of the differences in the organic and inorganic composition of these coals (Falcon 1986). Instead of using bituminous coal from the USA, we proposed to investigate South African coal waste (Eterigho-Ikelegbe et al. 2021b). The composites produced by the investigators (Hill and Easter 2021) using different South African coal wastes but with the same procedure and PCP showed that coal waste coded PCD (hydrogen/carbon ratio of 0.34) produced composites with the least desired properties. Specifically, the GTS coal waste manifested the highest hydrogen/carbon ratio of 0.72 and produced good quality composites with a flexural strength of 36.46 MPa and water absorption of 3.40%. In general, water absorption as low as 1.94 to 10.1% and flexural strength in the range of 27.51 to 36.46 MPa was obtained (Eterigho-Ikelegbe et al. 2021b). These values fulfil the requirement or standard specification expected of some building materials, such as ceramic tiles and clay roof tiles. The composites also displayed thermal stability in air and nitrogen atmosphere up to 600 °C and chemical resistance up to 99.97%. It was further shown that most of the carbon in the coal waste was trapped in composites.

This new, yet straightforward technological concept of combining coal waste and PCP to produce composites is a great way to consume a large amount of coal waste. From a social and environmental perspective, this technology concept has several merits, including a reduction in natural resource consumption, a new resource base for building materials, and a reduction in the carbon footprint of coal waste. Furthermore, the concept advances the United Nations Sustainable Development Goals 9, 11, and 12 leading to 2030. Based on this background, this study sets forth to shed light on the bonding mechanism between coal waste and the preceramic polymer resin towards the production of carbon/ceramic composites. We investigated the textual properties of the composites compared to the raw coal samples and the surface property of the composites was evaluated using the adsorption of deionised water as the polar liquid to measure the contact angle. Raman spectroscopy was employed to systematically study the graphitisation degree of the carbon phase in the composites and to understand the composites’ electrical resistivity behaviour. Finally, using coal waste to produce composites might pose exposure risks of heavy metal elements to the environment and human health when disposed of. As such, the comparison of the leached concentrations of heavy metal elements from the composites to standard leachable concentration thresholds was documented.

## Materials and methods

### Materials

**Fig. 1:**
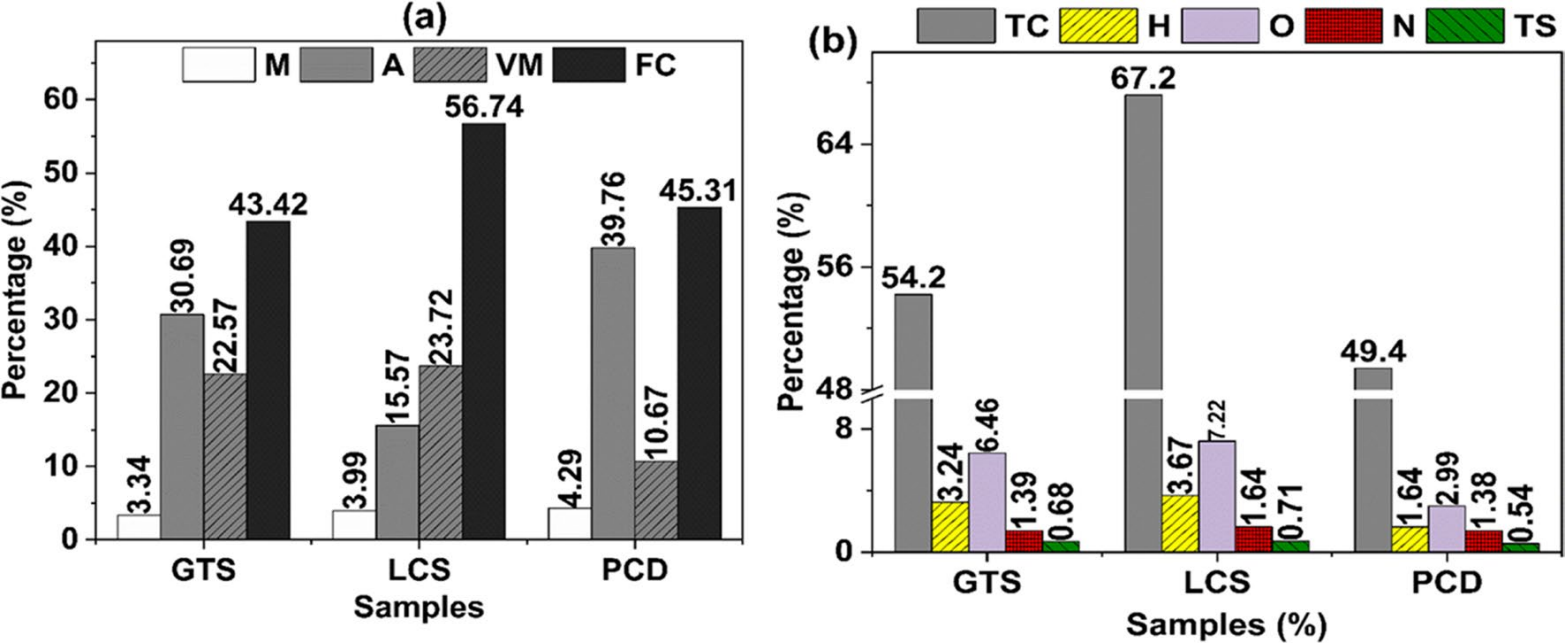
Assessment of the physicochemical characteristics of the coal wastes (air-dried). (**a**) Proximate analysis, (**b**) Ultimate analysis. *M*, inherent moisture; *A*, ash content; *V*, volatile matter; *FC*, fixed carbon; *TC*, total carbon; *TS*, total sulfur; *O*, oxygen; *O*, by difference as [100-(M + Ash + C + H + N + S)]; *FC*, by difference as [100-(VM + Ash + M)]

Coal wastes tagged GTS, PCD, and LCS were collected from the KwaZulu-Natal and the Witbank coalfields of South Africa. The assessment of the physicochemical characteristics of the coal wastes (Fig. 1) shows that PCD has the lowest volatile matter (10.67%) and highest ash content (39.76%) while LCS has the highest fixed carbon (56.74%). The particle size of the coal wastes is such that 80% is less than 45 µm. The ceramic-forming polymer used as the binder for producing the carbon/ceramic composites is a polysiloxane PCP specially formulated by XMAT®, the Advanced Materials Division of Semplastics (Florida, USA). The polysiloxanes are composed of repeated-silicon-oxygen- units (inorganic network) as the backbone and CH=CH_2_, H_2_, or CH_3_ as the organic constituents or side groups attached to the silicon atom.

### Preparation of the carbon/ceramic composites

**Fig. 2:**
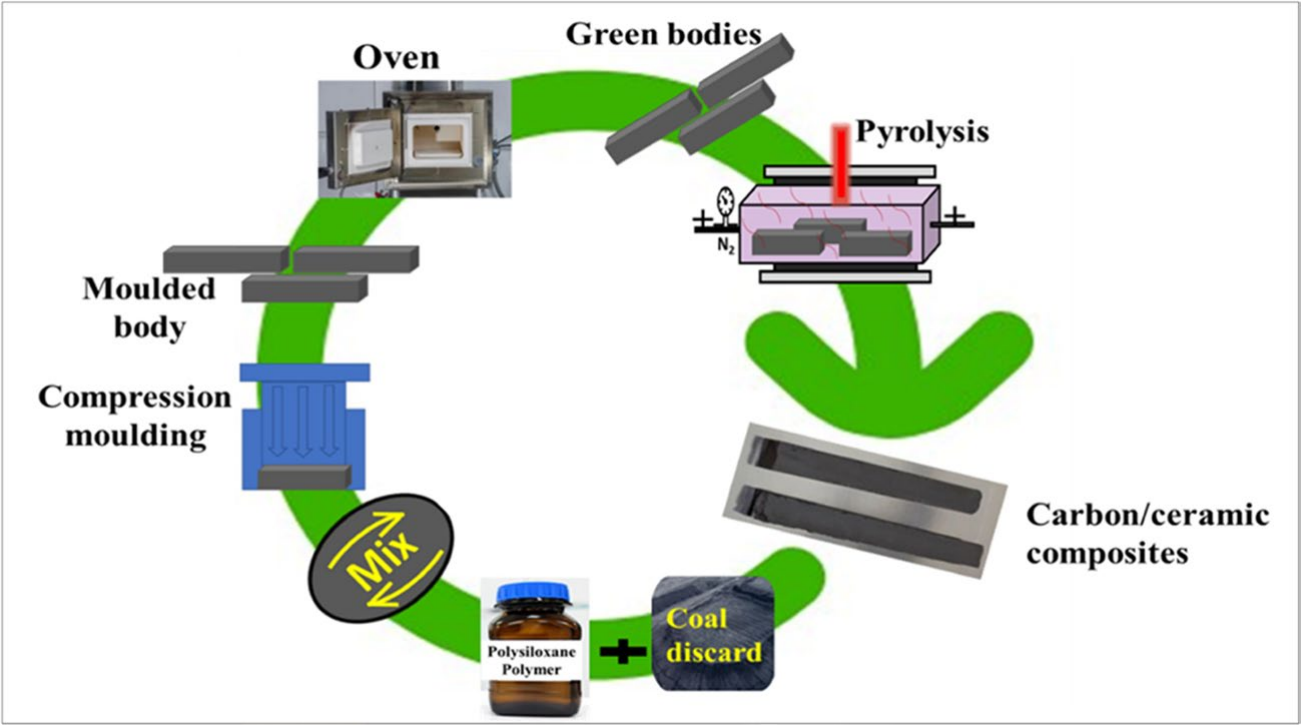
A summary of the production of the carbon/ceramic composites

**Fig. 3:**
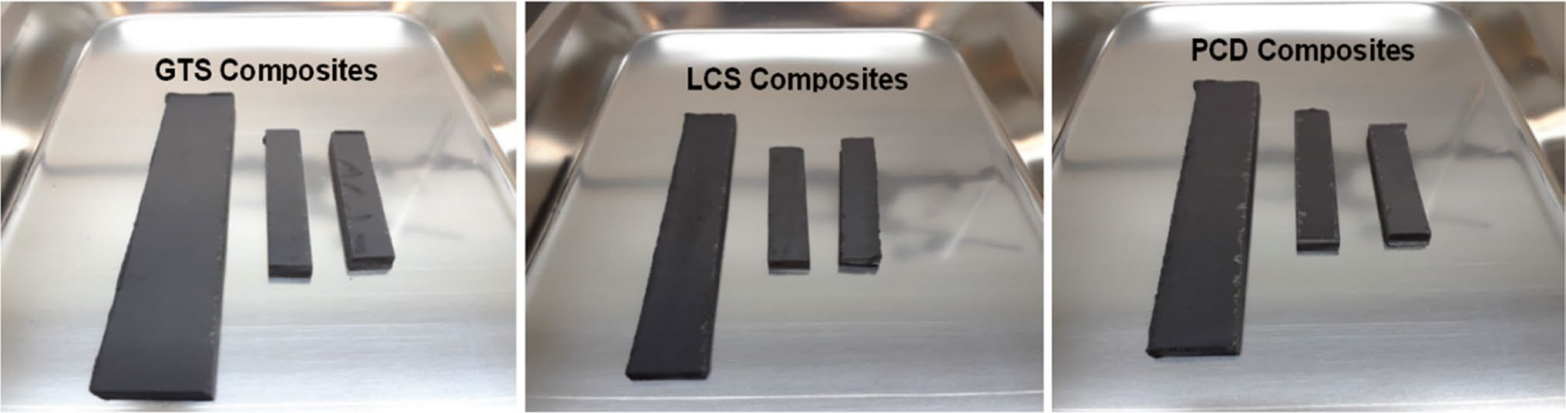
Pictorial images of the carbon/ceramic composites

The pulverised coal waste and the liquid PCP resin were sufficiently blended at a predetermined ratio in a mixer to wet the coal particles and fill the voids between the particles. The mouldable mixture is then uniaxially pressed into moulds like standard clay tiles to produce bodies with controlled geometries. The moulded bodies were cured in an oven at 150 °C and the cured bodies were pyrolysed in a retort furnace under a nitrogen atmosphere up to 1000 °C, maintaining the maximum temperature for 10 h. The furnace was allowed to cool to room temperature when the pyrolysis is complete and the cooled composites were analysed and tested. The detailed description of the production of the carbon/ceramic composites is presented in Eterigho-Ikelegbe et al. (2021b). Figure 2 displays a summary of the schematic production process of the carbon/ceramic composites and Fig. 3 shows the pictorial images of the produced carbon/ceramic composites.

### Characterisation and testing

#### Textural properties of the coal samples and carbon/ceramic composites

The evaluation of the textural properties of the crushed coal waste and the produced composites was based on Brunauer–Emmett–Teller (BET) nitrogen adsorption analyses of the degassed samples done at 77 Kelvin. An Autosorb iQ gas sorption instrument (Quantachrome Instruments, USA) with Quantachrome® ASiQwinTM software interfaces the Autosorb iQ to a computer to enable data acquisition and reduction. The pore size distribution was calculated from the nitrogen adsorption data based on the non-local density functional theory calculation model.

#### Wettability evaluation

The contact angle measurement device (OCA 15EC goniometer, DataPhysics instruments, GmbH, Germany) equipped with a microsyringe and a high-speed camera provided information about the wetting property (static contact angle) of the composite surface. The surface of the composite was dosed using deionised water at ambient conditions (dosing volume = 3 μL (microlitre); dosing rate = 2 μL/s (microlitre per second) from the syringe prior to measurement. Eight drops at different spots (top and reverse faces) of the composites were recorded after 10 s and the average value was reported.

#### Form of carbon/Raman analysis

The microcrystalline carbon structure, such as the degree of ordering and graphitisation of carbon in the coal and composites was characterised using Raman spectroscopy. Raman spectra were obtained for fine samples of the coal and composites using the excitation laser wavelength 514.5 nm (nm) line of the Lexel Model-95 SHG argon-ion laser. The instrument is integrated into a Horiba LabRAM HR Raman spectrometer fitted with an Olympus BX41 microscope and the data were acquired using LabSpec v.5 software.

#### Electrical resistivity measurement

**Fig. 4:**
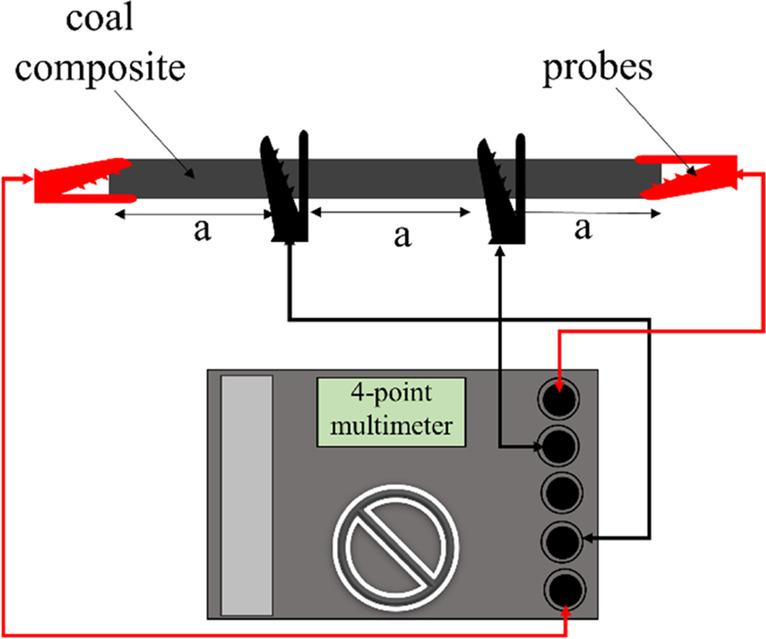
Arrangement of the four-point probe resistance measurement of the carbon/ceramic composites

The direct current (dc) volume resistance of the composite was measured using a multifunctional resistance analyser (1623-2 Earth Ground Tester, Fluke Corp., USA) in a four-point probe configuration, to gain insight into the electrical properties of the carbon/ceramic composites. The measurement range of the equipment for our four-point resistance is 0.02 ohms (Ω)-19.99 kiloohm (kΩ). A test voltage of 20 volts (V) was applied to measure the volume resistance of the composites. The probe was held firmly to the surface of the composite for 60 s and the distance between probes was separated by one-third of the total length of the composite (Fig. 4). The applied current comes in and out of the composites through the outer probes. The mean representative resistance from five electrical resistance measurements was used to calculate the bulk (volume) resistivity following Eq. (1).

1 $${R}_{C} =2\pi .a.{R}_{E}$$

where ${R}_{C}$ is the bulk or volume resistivity (Ω-cm) of the composites, $a$ is the probe distance (cm), and ${R}_{E}$ is the measured resistance (Ω).

#### Environmental friendliness of the composites

The end-of-life leaching behaviour of crushed composites was conducted under laboratory conditions using acetate buffer and deionised water as the eluants to determine the mobility of heavy metal elements. The eluant was added in a liquid-to-solid ratio of 20 mL/g (millilitre per gram) dry matter. The deionised water had a pH of 7.92 while an acetate buffer had a pH of 4.94. The acetate buffer pH was prepared based on Toxicity Characteristic Leaching Procedure (TCLP; US EPA Method 1311, 1992). 5.7 mL glacial acetic acid was added to approximately 900 mL of water to which 64.3 mL of 1 mol per litre sodium hydroxide solution was added. This solution was then diluted to 1 L with water and thoroughly mixed. The liquid/solid mixture prepared in screw cap bottles was continuously rotated at 30 revolutions per minute for 18 h using a roller mixer. Thereafter, the mixture was filtered over a 0.45 µm syringe filter to separate it from the solid phase. The pH value and the electrical conductivity of the eluates were recorded using the pH meter and conductivity meter (SevenCompact™ S220 and S230, Mettler-Toledo, GmbH). Inductively coupled plasma-mass spectrometry was subsequently used to determine the concentration of arsenic, barium, cadmium, chromium, lead, zinc, mercury, and selenium in the eluates. The concentrations of inorganic anions — chloride, fluoride, and sulphate were determined using ion chromatography. The results were then compared with the specified TCLP regulatory levels of heavy metal elements (US EPA 1992) and leachable concentration threshold (LCT) from the South African National Environmental Management Waste Act (NEMWA) (Molewa 2013). The end-of-life composite waste is considered hazardous if the determined concentrations of the metals in the eluates exceed both standard levels.

## Results and discussion

### Mechanism of bonding

As highlighted in the Materials and method section, the blend of coal waste and the polysiloxane polymer was pressed into a mould and thermally cured. Crosslinking via an addition mechanism of the different molecular, reactive, and/or functional groups in the blend is activated to yield a weakly bonded body during curing. At the early stage of pyrolysis (150 to 600 °C) of the cured composites under a nitrogen atmosphere, polycondensation and other molecular reactions occur between the coal particles and the PCP. This is accompanied by the release of some of the PCP’s side chain groups, hydroxyl structural groups in the raw materials, low molecular weight volatiles in coal, and the volatilisation of the silicon oligomers (Shibuya et al. 2007). As the pyrolysis progresses (at temperatures greater than 600 °C), the bonds in the coal particles are severed and polysiloxane degradation leads to a series of consecutive cleavage of the Si-O, Si-C, C-C, C=H, C-O, O-H and C-H bonds within the mixture. This causes complex recombination reactions between the silicon radicals and the reactive/decomposition products from the mixture. As the constituent of the coal undergoes vitrification and/or mineralisation, the organic components of the PCP transform into inorganic ceramic and graphitised carbon sandwiched into the coal’s carbon matrix to obtain dense carbon/ceramic composites. According to Hill and Easter (2021), the illite and kaolinite in the coals mineralise into silica and alumina nanoparticles during pyrolysis. Both the mineralisation (i.e., devolatilisation) and ceramisation process of the raw materials manifest as mass loss and dimensional shrinkage experienced by the body during pyrolysis.

**Fig. 5:**
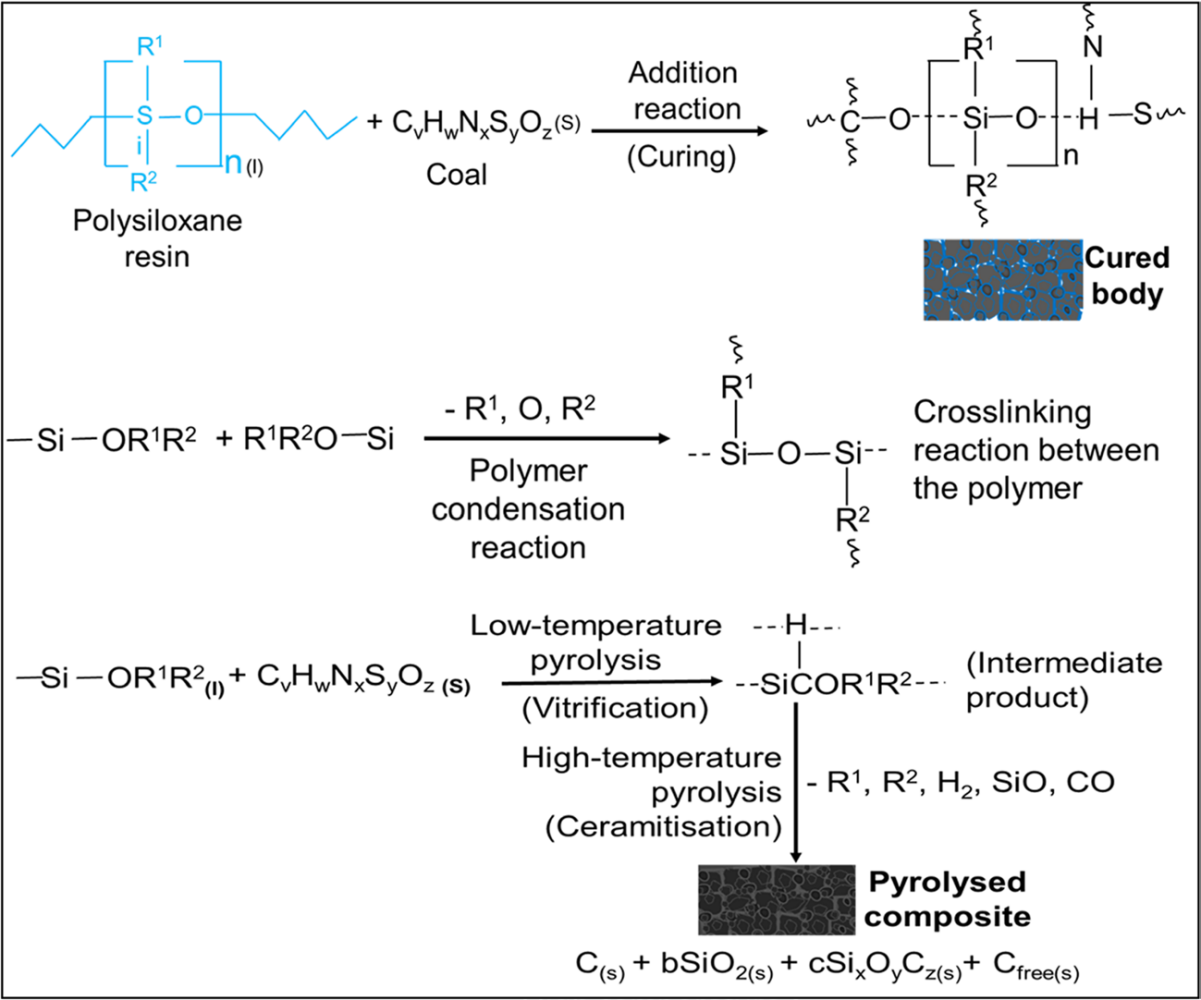
Hypothetical mechanisms of the curing and pyrolysis of coal waste and the polysiloxane preceramic polymer

The combination of reactions between the coal particles, the carbon, and other moieties of the polysiloxane polymer produces new phases and free carbon embedded in a carbon matrix. Most likely some silicon carbide nanocrystals and nanodomains of silicon dioxide that are surrounded by carbon domains could have been formed as pyrolysis of polysiloxanes is known to result in their growth (Pan et al. 2017; Shen et al. 2018). Figure 5 shows the hypothetical mechanism for composites production from coal waste and PCP resin. R^1^ and R^2^ are referred to as organic groups, such as methyl, phenyl, hydrogen, etc., bound to the silicon atom of the PCP. The polar and carbonyl groups in the coal waste react with these organic groups and other constituents of the PCP via hydrogen bonds during curing and pyrolysis to form the carbon/ceramic composites.

### Textural analysis of the coal samples and carbon/ceramic composites

**Fig. 6:**
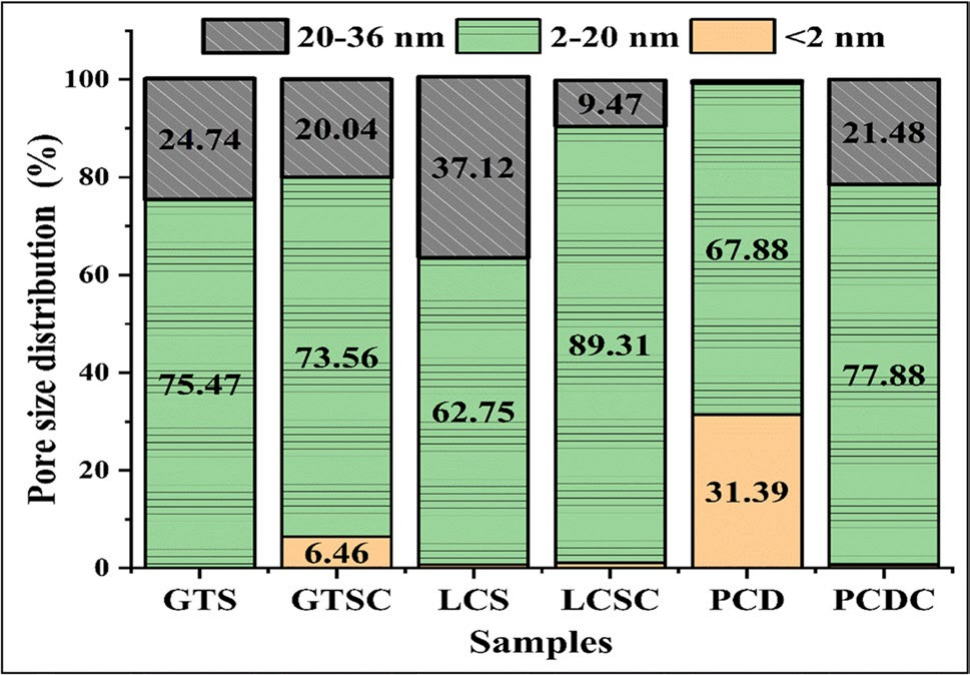
Comparison of the pore size distributions of the coal samples and the carbon/ceramic composites

Coal is a heterogeneous material with intrinsic organic and inorganic properties that can influence the textural properties of the composites produced. For instance, the pore network of coal could limit the mass transport, reactivity, and diffusivity between coal and the PCP during pyrolysis. Therefore, nitrogen gas physisorption measurements were conducted to characterise the textural properties of the coal samples and carbon/ceramic composites. Figure 6 displays the pore size distribution of the coal waste and the carbon/ceramic composite ceramics based on the density functional theory calculation model. Table 1 tabulates the Brunauer–Emmett–Teller surface area, average pore diameter, and total pore volume of the coal samples and respective carbon/ceramic composites.

**Table 1:** Textural information of the coal samples and the carbon/ceramic composites

Samples	BET SA (m^2^/g)	APD (nm)	TPV (cc/g)
GTS	9.791	11.56	0.019
LCS	5.174	18.08	0.013
PCD	18.113	3.34	0.014
GTSC	1.132	15.89	0.004
LCSC	2.268	6.12	0.003
PCDC	1.565	14.44	0.004

*APD*, average pore diameter; *BET*, Brunauer–Emmett–Teller; *GTSC*, GTS composite; *LCSC*, LCS composite; *PCDC*, PCD composite; *SA*, surface area; *TPV*, total pore volume based on non-local density functional theory model

From Fig. 6, GTS and LCS (average pore diameter of 11.56 and 18.09 nm) contain less than 0.5% micropore volume. The micropore volume of PCD around 31% (average pore diameter of 3.34 nm) suggests that PCD is from a high-rank coal dump and has a dense structure with low natural porosity and limited adsorption capacity. The dense nature of PCD waste might have impeded the flow of the PCP into the pores during mixing, thereby, limiting the diffusion and reaction between the PCD coal particles and PCP during pyrolysis. Furthermore, Lu and Do (1991) reported that during the pyrolysis of coal waste, volatiles are released. Analysis of the coal wastes indicated that the volatile matter content of GTS, LCS, and PCD is 22.57%, 23.72%, and 10.67%, respectively. As the pyrolysis of the coal/PCP blend progresses, the pores of GTS and LCS are prone to expand or even collapse as a result of volatile liberation. The resultant collapse of the pores might have enhanced the transport and reactivity of the PCP resin into these coal particles, facilitating bonding as the carbon/ceramic composites solidify into a rigid body. Apart from the physicochemical properties, it can be seen that the textural property of the coal waste is an important criterion to consider when selecting coal for this application. As expected, there is a significant decrease in the surface areas of the composites compared to the raw coals (Table 1) due to the rigidity of the composite particles. This suggests that the pyrolysis process adequately embedded and bound the ceramic-forming polymer with the coal particles.

### Water contact angle analysis

**Fig. 7:**
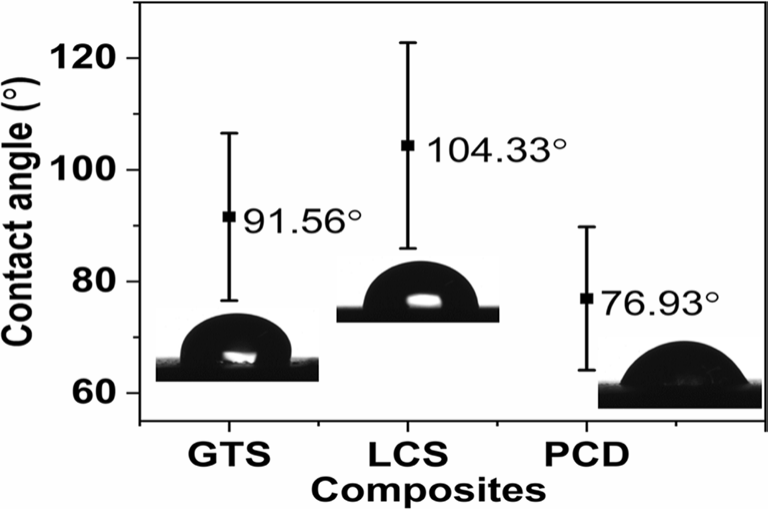
The water contact angle of the carbon/ceramic composites at room temperature

Water penetration significantly impacts the durability or degradation of construction products and engineering composites (Helmi and Hefni 2014; Li et al. 2021). Since the carbon/ceramic composites could be fabricated into composite building materials for outdoor applications, they must resist water penetration. From this standpoint, the water contact angle (CA) of the composites’ surface using deionised water as a polar solvent was measured to examine their hydrophobic performance. The shapes of the sessile drop on the composites (Fig. 7) indicate that the CA of the PCD composite is of average 76.93° ± 5 and could be considered hydrophilic (CA ˂ 90°). The contact angle of materials is inversely related to hydrophilicity and surface-free energy (Azimi and Asselin 2022). This implies that the surface-free energy of the PCD composite is high, hence, water-shedding on the surface is reduced, and the surface has a high tendency to wet or adsorb water. By contrast, the water CAs of the GTS and LCS composites of 91.56° and 104.33° suggest hydrophobic (CA ≥ 90°) surface properties, poor wettability, and the ability to decrease the rates of water adsorption (Duncan et al. 2005; Subedi 2011; Feyyisa et al. 2017). The relatively large error bar in Fig. 7 was attributed to the roughness of the composite surfaces. Specifically, the high CA of the GTS and LCS composites infers that they may not necessarily require secondary protection to prevent the ingress of water molecules that pose a threat to their degradation. Notable for conventional roofing materials (clay and concrete) that were also tested, it was difficult to capture CA images as the waterdrop spread on the surface and penetrated their pores quickly.

### Raman analysis

**Fig. 8:**
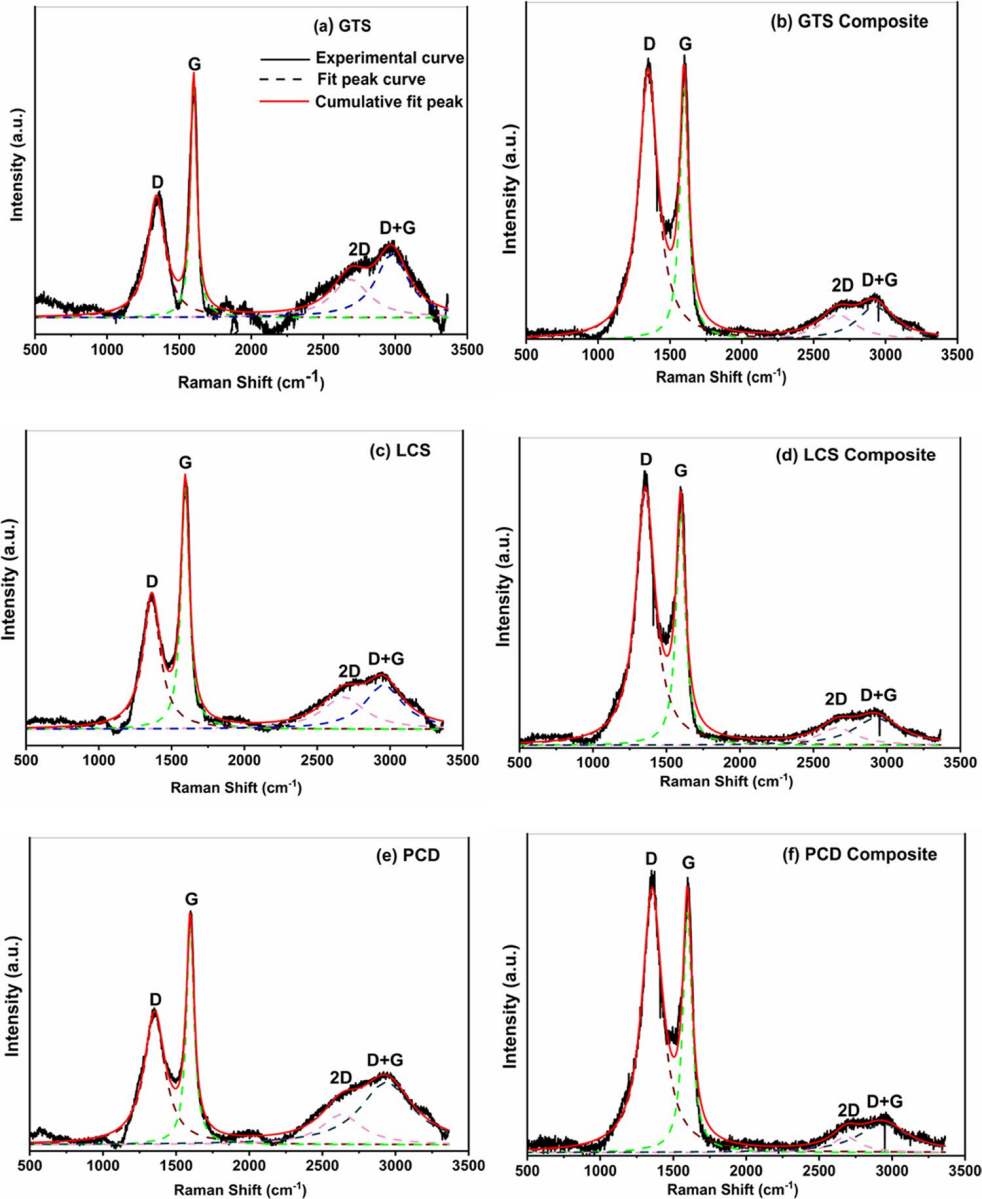
Raman spectra of the coal waste and the carbon/ceramic composites with their corresponding curve-fitting bands

Raman analysis was used to compare the structural state of the carbon atoms in the coal waste and that of the carbon/ceramic composites. The obtained spectra were deconvoluted (curve-fitted) using the Lorentz curve-fitting function (OriginPro 9.1.0 software, OriginLab Corporation, USA) based on the remarks of Angoni (1993). In principle, the structure of the crystallites of polycrystalline carbon materials is described by several parameters to correlate with the nanostructure of these materials. Equations (2) to (5) were employed to determine the quantitative spectral parameters, such as the peak position, peak intensity ratio $\left(\frac{{A}_{D}}{{A}_{G}}\right)$, size of carbon cluster crystallites $\left({L}_{a}\right)$, and other graphitisation indices (${L}_{D}$ and ${L}_{eq}$). The peak intensity ratio was estimated using the ratio of the integrated areas of the D (disordered) and G (graphitic) bands. *A*_*2D*_ represents the areas under the 2D peak. Graphitisation parameters of the carbon phase of the samples derived from the curve-fitted Raman spectrum are summarised in Fig. 8 and Table 2.

**Table 2:** Comparison of the other graphitisation indices obtained from deconvoluted Raman spectra of the coal samples and carbon/ceramic composites and calculated using Eq. (2) to (5)

Sample	Carbon content (%)	D-band (cm^−1^)	G-band (cm^−1^)	*L*_*D*_ (nm)	*n*_*D*_ [⨯10^11^](cm^−3^)	*L*_*eq*_ (nm)
GTS	56.70	1342	1600	9.49	4.79	5.63
LCS	68.55	1363	1594	10.70	3.77	4.89
PCD	51.40	1352	1597	8.95	5.39	3.77
GTS composites	40.00	1348	1598	7.41	7.86	1.47
LCS composites	42.70	1356	1597	7.49	7.71	1.11
PCD composites	44.05	1355	1597	7.33	8.03	0.70

2 $${L}_{a}=C{\lambda }_{L}{\left(\frac{{A}_{D}}{{A}_{G}}\right)}^{-1}\mathrm{nm}$$

3 $${L}_{eq}=8.8\left(\frac{{A}_{2D}}{{A}_{D}}\right)\mathrm{nm}$$

4 $${n}_{D}=\frac{2.4\times {10}^{22}}{{\lambda }_{L}^{4}}\left(\frac{{A}_{D}}{{A}_{G}}\right){\mathrm{cm}}^{-3}$$

5 $${L}_{D}=\sqrt{\left(1.8\times {10}^{-9}\right){\lambda }_{L}^{4}\left(\frac{{A}_{G}}{{A}_{D}}\right)\mathrm{nm}}$$

From Eq. (2), $C(514.5)=4.38 \mathrm{nm}$ (Ferrari and Robertson 2000; Jiang et al. 2009; Larouche and Stansfield 2010; Dalcanale et al. 2014). ${L}_{D}$ is the average distance between defects. ${L}_{eq}$, a graphitisation index introduced by Larouche and Stansfield (2010) describes the average continuous graphene length including tortuosity. This quantity gives an estimate of the equivalent phonon mean free path, provided complementary knowledge about the composites. Furthermore, the defect density, ${n}_{D}$, and the distance between defects, ${L}_{D}$, account for the amount of disorder and were calculated based on work presented in the literature (Cançado et al. 2011; Ricohermoso et al. 2022, 2023).

The first-order Raman spectrum of the coal wastes and carbon/ceramic composites (Fig. 7) exhibit two prominent features — the fingerprint D- and G-bands at Raman shifts between 1348 and 1363 cm^−1^, and 1594 and 1600 cm^−1^ found in many carbon materials (Ferrari and Robertson 2000). The D-band is associated with the vibration or breathing mode of the sp^2^ atoms in rings assigned to the A_1g_ modes observed in small crystallites or boundaries of larger crystallites of graphite; whereas the G-band, which stems from in-plane bond stretching of sp^2^ atoms in rings and chains, has been assigned to the *E*_*2g*_ modes observed in crystals of graphitic materials (Tuinstra and Koenig 1970; Ferrari and Robertson 2000; Sarkar et al. 2011; Dalcanale et al. 2014). Furthermore, two minor peaks corresponding to the 2D and D + G bands in the second-order Raman spectrum between 2400 and 3000 cm^−1^ can be observed in the coal and coal composite samples.

**Fig. 9:**
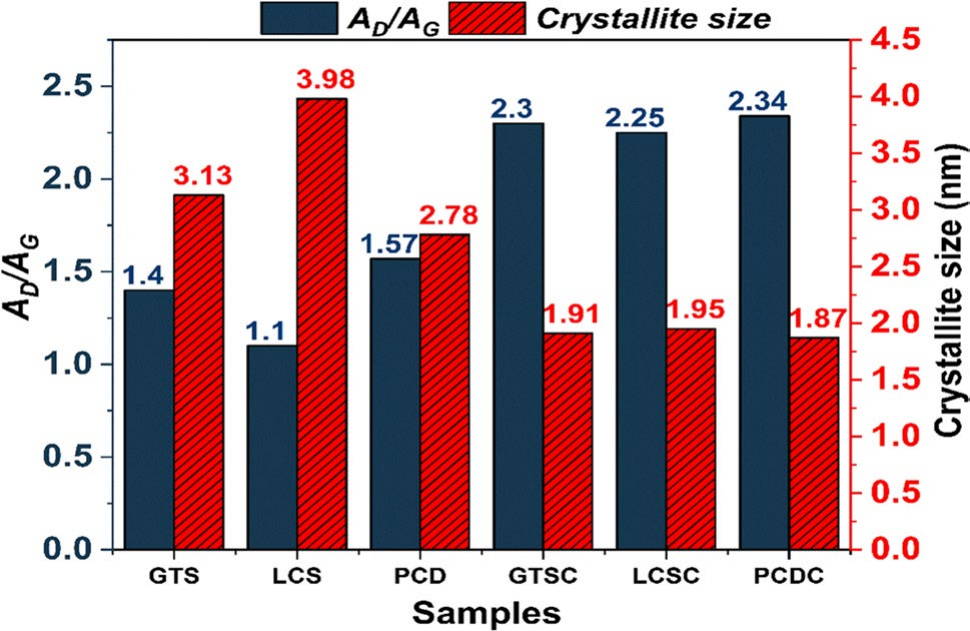
Variation in the peak area intensity ratio (*A*_*D*_*/A*_*G*_) and the size of the carbon cluster crystallites of the coal and their composites (*GTSC*, GTS composite; *LCSC*, LCS composite; *PCDC*, PCD composite)

The 2D band observed around 2700 cm^−1^ is usually found in defect free graphite samples while the D + G band is assigned to the disordered state of the carbon nanostructure in the samples (Gao et al. 2012; Colombo et al. 2010; Wen et al. 2020; Adigun et al. 2022). At close observation, this amorphous state decreases as the coal wastes transform into carbon/ceramic composites. Upon addition of PCP with the coal matrix and further curing/pyrolysis, the disorder degree of the carbon in the composites compared to the coal wastes increases judging from the calculated intensity ratio *A*_*D*_*/A*_*G*_ (Fig. 9), meaning there was a progressive conversion of sp^2^ carbon to sp^3^ carbon during pyrolysis. This confirms that the amorphous carbon structure (less organised carbon rings) is prevalent in the composites. In principle, the size of carbon crystallites, ${L}_{a}$ is reduced; hence, the inter-defect distance ${(L}_{D})$ reduces (Dalcanale et al. 2014) compared to the raw coal waste (see Fig. 9 and Table 2). Furthermore, a decrease in ${L}_{D}$ of the carbon phases invariably followed an increase in ${n}_{D}$ and a corresponding decrease in ${L}_{eq}$(Table 2). Comparing the composites, *A*_*D*_*/A*_*G*_ decreases from LCSC < GTSC < PCDC, reflecting that the LCS composite contains more graphitised carbon (He et al. 2017; Moni et al. 2017).

### Electrical resistivity evaluation

**Fig. 10:**
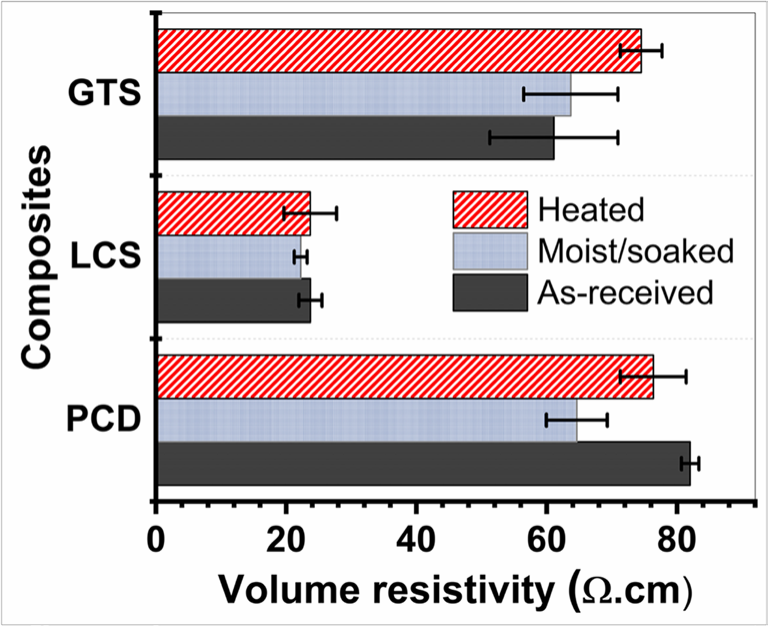
Measured volume electrical resistivity of the composites

The direct current (dc) volume resistivity of the composites (as-received, heated, and moist (soaked) composites) measured in a four-point probe configuration gave insight into how strongly or weakly the composites resisted the flow of current (Fig. 10). The standard deviation obtained from five measurements for each composite is less than 5.0% with volume resistivity in the range of 22 to 82 Ω-cm (4.4998 × 10^−2^ to 1.2194 × 10^−2^ Siemens per centimetre in terms of volume conductivity). The volume resistivity when the as-received composites were soaked in water for 3 days reduced. However, when heated under the same condition, there was no identifiable trend. Difficulty in maintaining the temperature of the composites in hot state may have led to the resistance being inaccurately measured, although great effort was taken to minimize these errors. The trend of volume resistivity of the composites at room temperature follows LCS < GTS < PCD. Interestingly, this is similar to the trend of porosity of the composites (LCS < GTS < PCD) but in reverse to the pyrolysis shrinkage (LCS˃GTS˃PCD) previously reported (Eterigho-Ikelegbe et al. 2021b).

According to Shibuya et al. (2007), the electrical resistivity of porous composites shows an increasing trend with porosity and is consistent with the composites produced in this study. Presumably, the level of shrinkage that occurred during pyrolysis reduced the interparticle distances and pushed the conducting atoms closer to themselves. Thereby, providing sufficient transport of mobile charge carriers resulting in the LCS composite manifesting the lowest electrical resistance.

Another explanation for the electrical resistivity behaviour of the composites can be drawn from features of the Raman spectra. The LCS composite has the lowest dc electrical resistivity compared to the GTS and PCD composites due to (i) lowest defect degree (i.e., highest graphitisation degree of sp^2^ hybridised carbon); (ii) highest carbon cluster size (1.95 nm), meaning the distance between the particles is smaller; thus, the percolation pathway for conduction is highly favoured (Xia et al. 2020; Ricohermoso et al. 2022). Ding et al. (2012) reported that high carbon size enhances the relaxation polarisation and does play a pivotal role in increasing the conductivity of the ceramic. Therefore, ${L}_{a}$ and the hybridization state of the carbon atom in the carbon/ceramic composite matrix are believed to exert an influence on the behaviour of the dc volume resistivity of the carbon/ceramic composites.

In general, there is no particular obligation on the electrical resistance performance of building materials. However, the ASTM D257-07 (2021) standard describes a solid material having a volume resistivity between 1 and 10,000,000 Ω-cm as moderately conductive. Beyond the physicomechanical properties, other considerations for building materials include dealing with heat build-up, lightning strikes, potential ignition, or explosion risk that arise from the electrostatic charge accumulation on the material surface (Aisenbrey 2007). As such, in areas where lightning strikes are prevalent, many conventional building materials grouped as insulators are limited in providing pathways for the dissipation of electrical charge accumulation. In fact, in the field of conductive polymer composites, low-volume resistivity composites are least susceptible to static electricity accumulation (Stabik and Chomiak 2016; Polok-Rubiniec et al. 2021). Moreover, the intrinsic electrically conductive network of carbon-based materials inspires a new generation of smart modern engineering materials. As a result, it is possible to deploy them as heat-generating elements, lightning strike shielding, and electromagnetic wave shielding (Shintani and Nakamura 1995; Fiala et al. 2017; Fu and Yuan 2017; Liu et al. 2022a, b). On the premise of their low-volume resistivity (22 to 82 Ω-cm), these composites may not be affected by electrostatic discharge and can be targeted for cold or icy environments to create self-heating parts that prevent ice build-up or help de-ice (Hill et al. 2022).

### Leachability study

Coal contains trace amounts of heavy metal elements liable to biological and/or chemical reactions with the environment. Also, the release of soluble constituents from the composites upon contact with liquid may pose a potential risk to the environment during their end-of-life. Therefore, it is important to understand the properties and risks of the composites when disposed of as crushed waste to the environment (end-of-life). The final pH and electrical conductivity of the eluates of the composites subjected to leaching in distilled water (pH 7.92) and acetate buffer solution (pH 4.94) are presented in Table 3.

**Table 3:** pH and electrical conductivity of the leachate

Composites	Distilled water		Acetate buffer	
	pH	EC (µS/cm)	pH	EC (µS/cm)
GTS	10.85 ± 0.3	33.32 ± 2.05	5.345 ± 0.015	568.45 ± 6.05
LCS	10.95 ± 0.34	39.45 ± 5.83	5.12 ± 0	541.4 ± 5
PCD	10.36 ± 1.12	48.62 ± 5.14	5.205 ± 0.005	560 ± 7

*EC*, electrical conductivity

It was observed that the pH values of the eluates based on the distilled water leaching protocol are in the range of 10.55 to 11.48, whereas the eluates obtained using the Toxicity Characteristic Leaching Procedure based on acetate buffer show a slightly acidic pH ranging between 5.36 and 5.12. In general, the pH of the eluates has slightly increased by an average of 37% for distilled water and 7% for the acetate buffer solution. It can be concluded that the pH of the eluants influences the final pH values of the eluates after the composites have been through leaching. The electrical conductivity of the eluates from the acetate buffer was found to be about 13 to 14 times higher than that of distilled water, suggesting the high mobility of ions from the crushed composites into the acetate buffer phase. Table 4 displays a snapshot of the leachable concentration of the heavy metal elements — arsenic, barium, cadmium, chromium, lead, zinc, mercury, selenium, and inorganic anions — fluoride, chloride, and sulphate from one stage batch test. From the Table, the leached concentrations of these heavy metals from the composites using deionised water and acetate buffer solution are below the TCLP regulatory levels of heavy metal elements (US EPA 1992). Therefore, the composite wastes could be considered non-hazardous at the end of life.

**Table 4:**
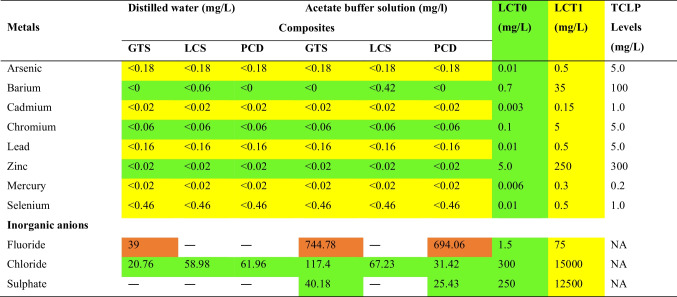
Leached concentrations of heavy metal elements and inorganic anions from the composites compared with the LCT and TCLP limits

*LCT*, leachable concentration threshold; *TCLP*, toxicity characteristic leaching procedure; *NA*, not available; **Type 3 waste**: *LCT0 < LC ≤ LCT1,* very low-risk hazardous waste with low potential for contaminant release and must be disposed of on a **Class C** containment barrier design landfill. **Type 2 waste:** *LCT1 < LC ≤ LCT2,* Considered low-risk waste with some potential for contaminant release and must be disposed of on a **Class B** containment barrier design landfill. **Green** indicates that the concentration of the waste material is below or equal to *LCT0*. **Yellow** indicates that the concentration of the waste material is above *LCT0* but below *LCT1*. **Brown** indicates that the concentration is above *LCT1* but below *LCT2*

Based on the South African classification system, arsenic, cadmium, lead, mercury, and selenium are above LCT0 but below LCT1 (LCT0˂LC ≤ LCT1). As such, the composite waste could be classified as Type 3 waste (see note below Table 4). In principle, the low concentration of these heavy metals in the eluate suggests that the pyrolysis of coal and the PCP into composites may have chemically rearranged these metals into stable phases. From Table 4 also, it can be seen that the release potential of these metals seemed to have not been influenced by the pH of the extraction fluids. However, the inorganic anions (fluoride, chloride, and sulphate) were influenced by pH. Acidic eluant resulted in more mobility of fluoride, chloride, and sulphate, in particular, fluoride, implying that these anions are highly soluble in an acidic solution. In general, when the composites are disposed of as crushed material in landfills, the waste falls between Type 2 and Type 3 waste and can be considered as low to very low hazardous risk waste. The leaching method reported in this study represents the leaching of heavy metals from the crushed composites assuming end-of-life. Overall. the results show that the leaching of these metals is unlikely to endanger the environment. However, this end-of-life approach may be inadequate for assessing the long-term environmental impact of the composites (i.e., use stage). Therefore, the utilisation scenario of the composites based on the tank leaching experiment designed for monolithic specimens should be conducted to estimate the leached concentration of heavy metal elements over a long period.

## Conclusion

This research involved investigating the bonding mechanism, textural properties, wettability, electrical behaviour, and the environmental friendliness of composites produced from coal waste and preceramic polymer resin. The evaluation concludes that the textural properties of coal waste (PCD) with the lowest volatile matter (10.67%) and highest ash content (39.76%) possessed a dense structure. This impeded the reactivity or bonding of the coal particles with the PCP resin during pyrolysis leading to low-quality PCD composites. The water contact angles of LCS and GTS composites were found to be above 90°, as such, the surface of these composites could be considered hydrophobic. The potential of these composites to reduce water penetration when used for outdoor applications is necessary for building materials. The *A*_*D*_*/A*_*G*_ ratios of the composites were higher than the coal wastes, indicating that the composites contain more amorphous carbon structures. The dc volumetric resistivity of the composites between 22 and 82 Ω-cm, classified as “moderately conductive”, was attributed to the presence of a percolating carbon network in the composites. As a result, the composites may not be prone to static charge accumulation responsible for causing fire hazards. Finally, acceptable levels of heavy metal elements in the eluates of the leached crushed composites suggest that the metals in the coal may have been chemically rearranged into stable phases during pyrolysis with the preceramic polymer resin. Based on the conclusions of the analyses presented in this article, this technique endows coal waste with a beneficial use as raw material to produce carbon/ceramic composites for building applications.

## Data Availability

No supplementary data or materials are available.
